# In Vivo Serotonin 5-HT2A Receptor Availability and Its Relationship with Aggression Traits in Healthy Individuals: A Positron Emission Tomography Study with C-11 MDL100907

**DOI:** 10.3390/ijms242115697

**Published:** 2023-10-28

**Authors:** Jeong-Hee Kim, Hang-Keun Kim, Young-Don Son, Jong-Hoon Kim

**Affiliations:** 1 Neuroscience Research Institute, Gachon University, Incheon 21565, Republic of Korea; 2 Biomedical Engineering Research Center, Gachon University, Incheon 21936, Republic of Korea; 3 Department of Biomedical Engineering, College of IT Convergence, Gachon University, Seongnam-si 13120, Republic of Korea; 4 Department of Psychiatry, Gachon University College of Medicine, Gil Medical Center, Gachon University, Incheon 21565, Republic of Korea

**Keywords:** aggression, serotonin, 5-HT2A receptor, positron emission tomography, C-11 MDL100907

## Abstract

Serotonergic neurotransmission has been associated with aggression in several psychiatric disorders. Human aggression is a continuum of traits, ranging from normal to pathological phenomena. However, the individual differences in serotonergic neurotransmission and their relationships with aggression traits in healthy individuals remain unclear. In this study, we explored the relationship between 5-HT2A receptor availability in vivo and aggression traits in healthy participants. Thirty-three healthy participants underwent 3-Tesla magnetic resonance imaging and positron emission tomography (PET) with [^11^C]MDL100907, a selective radioligand for 5-HT2A receptors. To quantify 5-HT2A receptor availability, the binding potential (BP_ND_) was derived using the basis function implementation of the simplified reference tissue model, with the cerebellum as the reference region. The participants’ aggression levels were assessed using the Buss–Perry Aggression Questionnaire. The voxel-based correlation analysis with age and sex as covariates revealed that the total aggression score was significantly positively correlated with [^11^C]MDL100907 BP_ND_ in the right middle temporal gyrus (MTG) pole, left fusiform gyrus (FUSI), right parahippocampal gyrus, and right hippocampus. The physical aggression subscale score had significant positive correlations with [^11^C]MDL100907 BP_ND_ in the left olfactory cortex, left orbital superior frontal gyrus (SFG), right anterior cingulate and paracingulate gyri, left orbitomedial SFG, left gyrus rectus, left MTG, left inferior temporal gyrus, and left angular gyrus. The verbal aggression subscale score showed significant positive correlations with [^11^C]MDL100907 BP_ND_ in the bilateral SFG, right medial SFG, left FUSI, and right MTG pole. Overall, our findings suggest the possibility of positive correlations between aggression traits and in vivo 5-HT2A receptor availability in healthy individuals. Future research should incorporate multimodal neuroimaging to investigate the downstream effects of 5-HT2A receptor-mediated signaling and integrate molecular and systems-level information in relation to aggression traits.

## 1. Introduction

Serotonergic neurotransmission has been implicated in impulsive and aggressive behaviors in animals and humans [1,2,3,4]. Most animal studies have reported that serotonergic neurotransmission is involved in the inhibitory control of interspecific or intraspecific aggression processes and behaviors [1,2]. In clinical studies, patients with personality disorders (PD) characterized by impulsivity and aggressiveness, i.e., borderline PD and antisocial PD, and those with intermittent explosive disorder showed significantly altered serotonergic neurotransmission, as revealed by positron emission tomography (PET) neuroimaging [5,6,7,8]. Collectively, the studies on patients with these specific PDs generally show findings supportive of perturbed serotonergic signaling being associated with pathologically increased impulsive and aggressive behaviors, although several studies including patients with past major depression [9,10] and comorbid alcohol dependence [11] did not find such associations. Interestingly, a recent comprehensive postmortem study of serotonergic markers in subjects with antisocial behaviors demonstrated significantly increased levels of serotonin 5-HT2A receptors but not of 5-HT1A, 5-HT1B, 5-HT2C, or 5-HT4 receptors [12].

Although aggressive behaviors are a common feature of borderline PD, antisocial PD, and several other psychiatric disorders, even healthy individuals can be at increased risk of such behaviors under certain conditions, such as when hatred and resentment are fueled by a variety of factors, e.g., economic inequality [13], interpersonal conflicts [14], and media exposure [15,16,17]. Previous studies have reported that symptoms in non-clinical groups that exhibit specific personality traits but do not fully meet the criteria for PDs, while they may not be as severe as those noted in PD groups, may still exhibit qualitatively similar traits [18,19]. Hence, based on these previous studies, aggressive behaviors are considered a continuum of traits, ranging from normal to pathological phenomena [20].

To date, individual differences in serotonergic neurotransmission in vivo and their relationships with aggression in healthy participant groups have been investigated in a few PET molecular imaging studies of 5-HT1A [21,22], 5-HT2A [23], and 5-HT4 [24] receptors. These studies have yielded some inconclusive results, although intra-synaptic serotonin deficiency was implied in studies showing positive correlations between postsynaptic serotonin receptor availability and aggression levels [22,24]. Several genetic association studies have shown relationships between 5-HT2A receptor polymorphisms and aggression traits in healthy individuals [25,26].

Aggression traits are multidimensional and composed of several subcomponents, such as physical aggression, verbal aggression, anger, and hostility [27]. These traits encompass affective, cognitive, and behavioral aspects. However, few studies on the relationship between serotonergic neurotransmission using PET and aggression traits in healthy individuals have investigated these separate subcomponents of aggression traits and their relationships with in vivo serotonergic markers.

Therefore, we aimed to explore the relationship between 5-HT2A receptor availability in vivo using PET with [^11^C]MDL100907, a radioligand that provides both high selectivity for 5-HT2A receptors and a high signal-to-noise ratio, and aggression traits (physical aggression, verbal aggression, anger, and hostility) in healthy individuals. We chose 5-HT2A receptor availability based on the findings of a recent postmortem study on comprehensive serotonergic markers in subjects with impulsive and aggressive behaviors [12] and preclinical studies in which 5-HT2A receptor modulation, particularly antagonism, demonstrated anti-aggressive effects in rodent models of aggression [28,29,30].

## 2. Results

The mean scores for the physical aggression, verbal aggression, anger, and hostility subscales were 13.3 ± 2.5, 9.0 ± 2.8, 9.6 ± 2.3, and 10.8 ± 2.2, respectively, and the mean total aggression score was 42.6 ± 6.5 (Table 1). The aggression scores were not significantly correlated with age (*r* = −0.13 to 0.21, *p* > 0.05). No significant sex differences were found in the verbal aggression, anger, and hostility subscale scores or in the total score (t = −0.74 to 1.78, *p* > 0.05); however, the physical aggression subscale score was significantly higher in males than in females (t = 3.31, *p* = 0.002).

**Figure 1:**
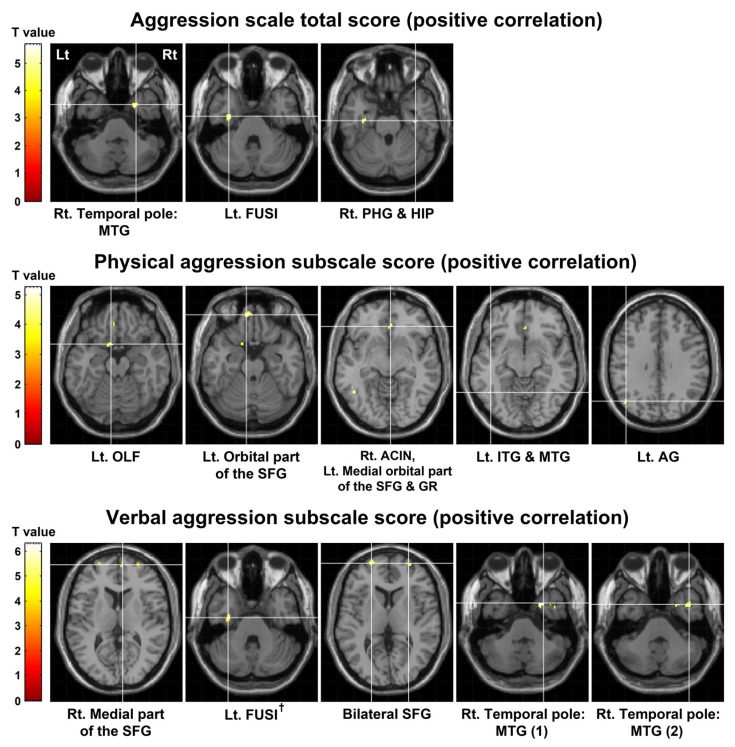
Results of voxel-based correlation analysis between the aggression scores and [^11^C]MDL100907 BP_ND_. These results are shown at a peak-level threshold of uncorrected *p* < 0.001 with an extent threshold of 20 voxels. ^†^ The verbal aggression subscale score also showed a significant positive correlation with [^11^C]MDL100907 BP_ND_ in the left FUSI at a peak-level threshold of FDR-corrected *p* < 0.05 with an extent threshold of 10 voxels (FDR-corrected *p* = 0.028, k = 12 voxels). BP_ND_, binding potential with respect to non-displaceable compartment; FUSI, fusiform gyrus; FDR, false discovery rate; Lt, left; Rt, right; MTG, middle temporal gyrus; PHG, parahippocampal gyrus; HIP, hippocampus; OLF, olfactory cortex; SFG, superior frontal gyrus; ACIN, anterior cingulate and paracingulate gyri; GR, gyrus rectus; ITG, inferior temporal gyrus; AG, angular gyrus.

**Figure 2:**
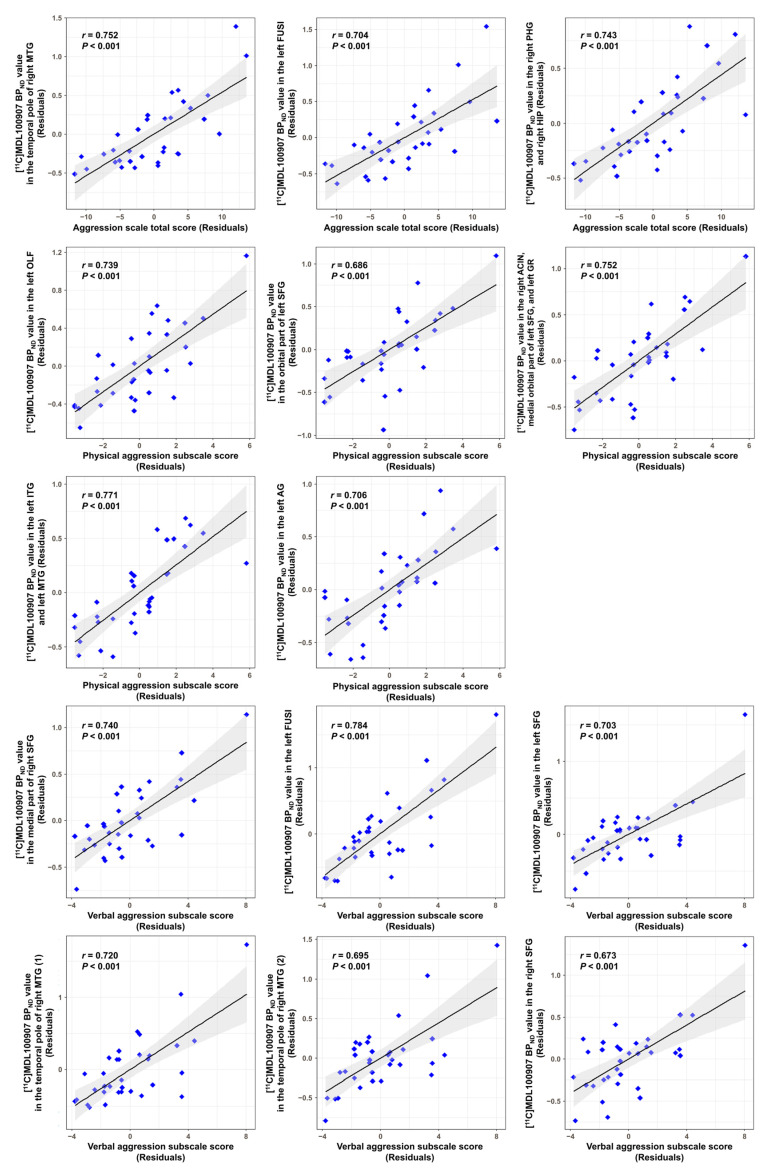
Scatter plots showing the correlations between the aggression scores and [^11^C]MDL100907 BP_ND_ in the clusters that showed statistical significance in voxel-based correlation analysis. The blue rhombi indicate ordered pairs of the unstandardized residuals estimated from two separate linear regressions of the aggression scores and [^11^C]MDL100907 BP_ND_ in clusters in regard to age and sex. The solid lines and gray areas represent the regression lines and 95% confidence intervals, respectively. BP_ND_, binding potential with respect to non-displaceable compartment; MTG, middle temporal gyrus; FUSI, fusiform gyrus; PHG, parahippocampal gyrus; HIP, hippocampus; OLF, olfactory cortex; SFG, superior frontal gyrus; ACIN, anterior cingulate and paracingulate gyri; GR, gyrus rectus; ITG, inferior temporal gyrus; AG, angular gyrus.

The voxel-based correlation analysis with age and sex as covariates revealed that the total aggression score was significantly positively correlated with [^11^C]MDL100907 binding potential with respect to non-displaceable compartment (BP_ND_) in the temporal pole of the right middle temporal gyrus (MTG) (uncorrected *p* < 0.0001), left fusiform gyrus (FUSI) (uncorrected *p* < 0.0001), right parahippocampal gyrus (PHG) (uncorrected *p* = 0.0001), and right hippocampus (HIP) (uncorrected *p* = 0.0001). The physical aggression subscale score had significant positive correlations with [^11^C]MDL100907 BP_ND_ in the left olfactory cortex (uncorrected *p* < 0.0001), orbital part of the left superior frontal gyrus (SFG) (uncorrected *p* < 0.0001), right anterior cingulate and paracingulate gyri (uncorrected *p* < 0.0001), medial orbital part of the left SFG (uncorrected *p* = 0.0001), left gyrus rectus (GR) (uncorrected *p* = 0.0003), left inferior temporal gyrus (ITG) (uncorrected *p* < 0.0001), left MTG (uncorrected *p* < 0.0001), and left angular gyrus (AG) (uncorrected *p* = 0.0001). The verbal aggression subscale score was significantly positively correlated with [^11^C]MDL100907 BP_ND_ in the medial part of the right SFG (uncorrected *p* < 0.0001), left FUSI (false discovery rate (FDR)-corrected *p* = 0.028), bilateral SFG (left: uncorrected *p* < 0.0001, right: uncorrected *p* = 0.0001), and temporal pole of the right MTG (uncorrected *p* < 0.0001). The scores on the anger and hostility subscales did not show significant correlations with [^11^C]MDL100907 BP_ND_ at the significance threshold. Details of the results are presented in Figure 1 and Table 2. Moreover, scatter plots showing the correlations between the aggression scores and [^11^C]MDL100907 BP_ND_ values in the clusters with statistical significance are shown in Figure 2.

We further conducted a supplementary region-of-interest (ROI)-based correlation analysis between the aggression scores and [^11^C]MDL100907 BP_ND_ in automated anatomical labeling (AAL) [31] atlas-based brain regions containing clusters that reached statistical significance in the voxel-based correlation analysis. The analysis showed that the total aggression score was positively correlated with [^11^C]MDL100907 BP_ND_ in the temporal pole of the right MTG (*r* = 0.447, *p* = 0.012) and right HIP (*r* = 0.429, *p* = 0.016). The physical aggression subscale score had positive correlations with [^11^C]MDL100907 BP_ND_ in the orbital part of the left SFG (*r* = 0.425, *p* = 0.017), medial orbital part of the left SFG (*r* = 0.387, *p* = 0.032), and left GR (*r* = 0.444, *p* = 0.012). The verbal aggression subscale score also showed positive correlations with [^11^C]MDL100907 BP_ND_ in the temporal pole of the right MTG (*r* = 0.382, *p* = 0.034). No other correlations were observed at *p* < 0.05. Details of these results are shown in Supplementary Figure S1.

## 3. Discussion

In this study, we found that human aggression traits were significantly positively associated with 5-HT2A receptor availability, particularly in the prefrontal cortical regions. Notably, physical and verbal aggression traits had significant positive correlations with 5-HT2A receptor availability in the orbital and medial prefrontal regions. These results are in line with the findings of a recent comprehensive postmortem study that reported significant elevations of 5-HT2A receptor levels in the orbital prefrontal cortex of individuals with antisocial behaviors [12]. Our results also support previous findings showing significant positive correlations between irritability scores and prefrontal 5-HT2A receptor availability in intermittent explosive disorder [6]; however, our participants were all healthy individuals who did not exhibit pathologically increased aggression.

Our in vivo PET molecular imaging findings suggest that altered serotonergic neurotransmission in higher cortical regions, especially in the prefrontal cortex, may play a significant role in aggression traits in healthy individuals. These findings are in accordance with the results of genetic association studies, which revealed that 5-HT2A receptor polymorphisms in the promoter and coding regions are associated with aggressive behaviors [26,32,33] and that 5-HT2A receptor polymorphisms associated with aggression showed an increased number of 5-HT2A receptor binding sites in the brain [34] and blood [35].

We also found significant positive correlations between human aggression traits and 5-HT2A receptor availability in the right HIP and right PHG. The HIP is one of the brain regions that make up the aggression-related neural network [36], which plays an important role in the regulation of aggressive behaviors [37,38]. Several clinical studies have demonstrated hippocampal dysfunction in subjects with antisocial and violent behaviors [38,39], and two PET studies have shown elevated hippocampal 5-HT2A receptor levels in patients with borderline PD [40,41]. The medial temporal lobe, which includes both the HIP and PHG, has also been shown to exhibit metabolic abnormalities in individuals with aggression [42]. The temporal pole of the MTG and ITG is also one of the brain regions involved in aggressive behaviors [43] and has been linked to vulnerability to violence and aggression [44]. This relationship is supported by previous clinical studies demonstrating aggressive behaviors in patients with tumors in the temporal lobe [45] and temporal lobe epilepsy [46]. In addition to these previous reports, our PET molecular imaging study suggests that 5-HT2A receptor availability in the temporal lobe may play an important role in human aggression.

Notably, the verbal aggression subscale score was significantly positively correlated with 5-HT2A receptor availability in the FUSI at an FDR-corrected threshold of *p* < 0.05. This finding is in accordance with the results of a previous functional MRI study using a script-driven imagery task, which demonstrated a large neural network associated with aggression, encompassing the prefrontal cortices, superior and middle temporal cortices, cingulate cortices, superior parietal cortex, middle occipital cortex, insula (INS), HIP, thalamus, and FUSI [47]. Our results support the notion that aggression-related behaviors are associated with neural connectivity in regions of cognitive control, affective salience, and visual networks, suggesting that 5-HT2A receptor-mediated signaling may play important roles in this circuitry.

The significant positive correlations between aggression traits and in vivo 5-HT2A receptor availability in our study are not in line with the findings of a previous PET study that showed no significant associations between frontal 5-HT2A receptor binding and aggression or impulsivity in healthy individuals [23]. Differences in the age range, ethnicity, rating scales, and radioligands used may account for the different results. Further molecular PET studies of the 5-HT2A receptors are warranted to confirm the involvement of 5-HT2A-mediated neurotransmission in aggression traits in diverse healthy populations.

The increased 5-HT2A receptor availability associated with high levels of aggression may be mediated via specific genetic polymorphisms [25,33,48], epigenetic factors [49], or regulation via neurotrophins such as brain-derived neurotrophic factors [50]. It is unclear whether increased postsynaptic 5-HT2A receptor availability reflects diminished synaptic serotonin levels. However, a previous PET study using a rapid tryptophan depletion paradigm demonstrated that [^11^C]MDL100907 binding does not compete with endogenous serotonin in humans [51]. In addition, as significant individual variations in the affinity of 5-HT2A receptors for the antagonist radioligand ([^11^C]MDL100907) are unlikely, the higher BP_ND_ in our study can generally be interpreted as being driven mainly by a higher Bmax and vice versa. In contrast, high postsynaptic 5-HT2A receptor binding may reflect compensatory upregulation owing to chronically low 5-HT2A receptor occupancy by low endogenous serotonin [52].

Previous PET imaging studies reported significantly elevated 5-HT2A receptor binding in patients with pathologically increased aggression [6,8]. Our results suggest that increased 5-HT2A receptor binding is also associated with human aggression personality traits in healthy individuals. Because 5-HT2A receptors are found in the cortical pyramidal cells, as well as in the subpopulation of inhibitory cortical interneurons, 5-HT2A receptors are involved in the regulation of the signal-to-noise ratio among cortical columns [53,54]. Hence, subtle changes in the 5-HT2A receptor density in the orbital prefrontal cortex may affect the assessment of internal and external stimuli [6]. Subsequently, in terms of neural circuitry, perturbed inhibitory prefrontal control over subcortical regions, including limbic areas, may induce exaggerated affective reactivity and emotional dysregulation, with effects such as enhanced aggression [3,4].

The interpretation of the results of this present study should be considered in light of certain limitations. Because personality traits may be driven by the composite effects of multiple genes and epigenetic factors, we note that a correlation between certain receptor availabilities and a personality trait does not mean that the neuroreceptor protein itself has a causal role or is a biomarker of that personality trait [55]. The range of aggression scores in healthy individuals is limited, which may limit the detection of significant correlations with 5-HT2A receptor availability [23]. Further, larger studies including patients with psychiatric disorders as the comparison groups and analyzing participants’ genetic polymorphisms in 5-HT receptors could provide a better interpretation of the current PET imaging results observed in healthy individuals. In our study, [^11^C]MDL100907 BP_ND_ was quantified using the basis function implementation of the simplified reference tissue model (SRTM); however, a two-tissue compartment model (2TCM) with arterial input function is optimal [51,56]. The arterial input kinetic model for 2TCM is measured using invasive methods such as radial artery cannulation, and the resulting discomfort might be a confounding factor and would also lead to limited recruitment of participants. Inaccuracies in determining the arterial input function can also be a source of bias in endpoint estimation [57]. Therefore, in our study, [^11^C]MDL100907 BP_ND_ values were obtained using the basis function implementation of the SRTM with the cerebellum as the reference region devoid of specific binding, as previously suggested for this tracer [51,58,59].

## 4. Materials and Methods

### 4.1. Participants

The study protocol was approved by the Institutional Review Board of the Gachon University Gil Medical Center, and all study procedures were conducted in accordance with international ethical standards and the Declaration of Helsinki. All participants were fully informed of the purpose and procedures of this study and provided written informed consent before participating in this study.

Thirty-three healthy participants (10 males and 23 females) with a mean age of 30.9 ± 8.3 years (range = 20–50 years) were enrolled in this study if they fulfilled all of the following criteria: (i) age between 19 years (legal adult age in South Korea) and 60 years, (ii) no past or current psychiatric disorders according to the Korean version of the Mini International Neuropsychiatric Interview (MINI) [60,61], (iii) no past or current substance dependence/abuse, (iv) no family history of psychiatric disorders, (v) no history of neurological or medical disorders, (vi) no past or current use of substances/medications known to affect the central nervous system, (vii) not pregnant on the date of the PET scan, and (viii) not meeting any exclusion criteria for magnetic resonance imaging (MRI) scan. The demographic and clinical characteristics of the participants are summarized in Table 1.

### 4.2. Clinical Assessment

Participants’ aggression traits were assessed using the Buss-Perry Aggression Questionnaire (BPAQ) [27]. The BPAQ is a 29-item questionnaire with each item rated on a 5-point scale from 1 to 5 and consists of four subscales: physical aggression, verbal aggression, anger, and hostility. Higher scores indicate more aggressive behaviors. In this study, we used the standardized Korean version of the BPAQ [62] and obtained individual subscale scores, as well as a total score.

### 4.3. Image Acquisition

All participants underwent scanning using a Biograph 6 PET scanner (Siemens Medical Imaging Systems, Knoxville, TN, USA) with [^11^C]MDL100907 (Neuroscience Research Institute, Gachon University, Incheon, Republic of Korea). A computed tomography-based transmission scan was conducted for attenuation correction prior to [^11^C]MDL100907 injection. After a bolus injection of 688.0 ± 52.5 MBq [^11^C]MDL100907 with an average molar activity of 65.0 ± 32.9 GBq/μmol (Table 1), emission data were obtained in dynamic mode for 90 min with 22 frames of the following durations: 4 × 30 s, 2 × 60 s, 2 × 90 s, 3 × 150 s, 3 × 210 s, 4 × 300 s, 3 × 600 s, and 1 × 900 s. The emission data were reconstructed using the two-dimensional ordered-subset expectation-maximization algorithm. The reconstructed PET frames had a voxel size of 1.33 × 1.33 × 1.50 mm^3^ and a matrix size of 256 × 256 × 109. These PET frames were corrected for attenuation, decay, detector dead time, random and scatter coincidences, and detector normalization.

Structural MRI data were obtained using a 3-Tesla MRI scanner (Magnetom Vida; Siemens Healthcare, Erlangen, Germany) with a three-dimensional T1-weighted magnetization-prepared rapid gradient echo sequence. This sequence entailed the following scan parameters: repetition time = 1800 ms, echo time = 2.61 ms, inversion time = 900 ms, flip angle = 10°, matrix size = 512 × 416, voxel size = 0.5 × 0.5 × 1.0 mm^3^, and number of slices = 176. During the PET and MRI scans, the participants’ heads were held as comfortably as possible, using sponges to minimize their head movement.

### 4.4. Image analysis

Image preprocessing was performed using Statistical Parametric Mapping 12 (SPM12; The Wellcome Centre for Human Neuroimaging, London, UK; www.fil.ion.ucl.ac.uk (accessed on 1 October 2014)). For motion correction within all reconstructed PET frames, realignment was performed according to a two-pass procedure. The first-pass realignment registered each frame to the first frame in the series. After this realignment, a mean PET image was computed. The second-pass realignment registered each frame from the first-pass alignment to the mean PET image. The structural MRI image was coregistered to the mean PET image derived from the realignment step using a 12-parameter affine transformation. Finally, the coregistered structural MRI images and the corresponding PET frames were spatially normalized using the Montreal Neurological Institute template.

**Figure 3:**
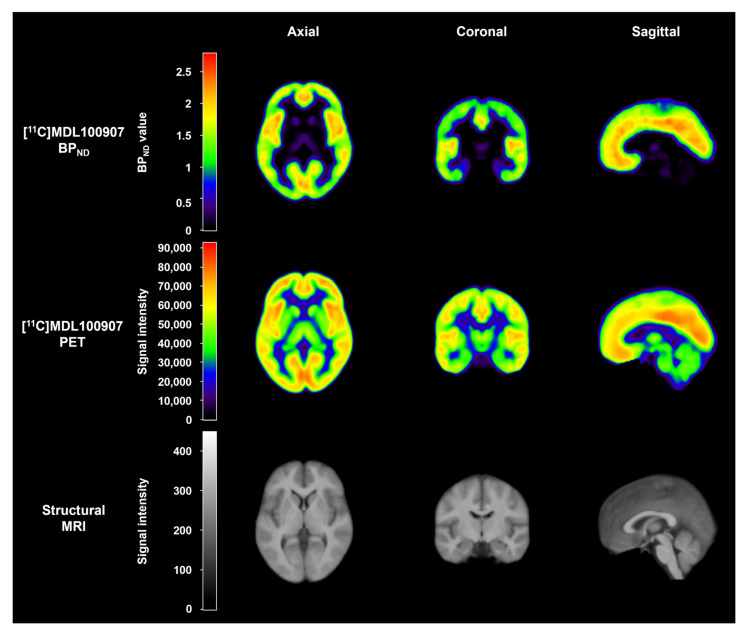
Representative mean images of [^11^C]MDL100907 BP_ND_, [^11^C]MDL100907 PET, and corresponding structural MRI in 33 healthy subjects. BP_ND_, binding potential with respect to non-displaceable compartment; PET, positron emission tomography; MRI, magnetic resonance imaging.

Based on the parameter estimation by kinetic modeling implemented in the PMOD software v4.2 (PMOD Technologies Ltd., Zürich, Switzerland), the BP_ND_ image of [^11^C]MDL100907 was obtained using a basis function implementation of the SRTM [51,63,64] with cerebellar gray matter as the reference region, as suggested in previous PET studies on this radioligand [51,58,59]. Representative mean images of [^11^C]MDL100907 PET and structural MRI are shown in Figure 3.

### 4.5. Statistical Analysis

Several studies have shown that age and sex influence aggression [65,66,67] and 5-HT2A receptor availability [68,69]. Our study also found a significant difference in the physical aggression subscale score between men and women (*p* = 0.002) (Supplementary Table S1). Furthermore, a voxel-based correlation analysis showed that age was negatively correlated with [^11^C]MDL100907 BP_ND_ in widespread regions, including the precentral gyrus (PRE), postcentral gyrus, Rolandic operculum, frontal lobe, parietal lobe, occipital lobe, temporal lobe, limbic lobe, and INS, at a peak-level threshold of uncorrected *p* < 0.005 with an extent threshold of 20 voxels (Supplementary Table S2). A voxel-based between-sex comparison analysis also showed that males had higher [^11^C]MDL100907 BP_ND_ in the left PRE, right AG, right middle frontal gyrus, right putamen, right MTG, left PRE, and left FUSI (all *p* values ≤ 0.005) compared to females, and females had higher [^11^C]MDL100907 BP_ND_ in the right calcarine gyrus and right cuneus (all *p* values ≤ 0.005) compared to males (Supplementary Table S3).

Therefore, to investigate the relationship between aggression traits and [^11^C]MDL100907 BP_ND_ in the brain regions of healthy individuals, we performed a voxel-based partial correlation analysis with age and sex as covariates using SPM12. As our study was exploratory in nature, without a priori hypotheses, significant results were defined at a peak-level threshold of uncorrected *p* < 0.001 with an extent threshold of 20 voxels, which has been used as a statistical significance threshold in previous PET studies [70,71,72].

## 5. Conclusions

Our findings suggest the possibility of positive correlations between aggression traits and in vivo 5-HT2A receptor availability, as measured using [^11^C]MDL100907 PET, in specific cerebral regions in healthy individuals. Future research should incorporate multimodal neuroimaging to investigate the downstream effects of 5-HT2A receptor-mediated signaling and integrate molecular- and system-level information in relation to aggression traits.

## Figures and Tables

**Table 1:** Demographic/clinical characteristics and [^11^C]MDL100907 PET scan information.

Variable	Mean (SD)/Number (%)
Demographic characteristics	
Age (years)	30.9 (8.3)
Male	33.3 (8.6)
Female	29.8 (8.2)
Sex	
Male	10 (30.3%)
Female	23 (69.7%)
Education (years)	15.6 (0.8)
Clinical characteristics	
BPAQ	
Physical aggression	13.3 (2.5)
Verbal aggression	9.0 (2.8)
Anger	9.6 (2.3)
Hostility	10.8 (2.2)
Total	42.6 (6.5)
[^11^C]MDL100907 PET scan information	
Injected dose (MBq)	688.0 (52.5)
Specific activity (GBq/μmol)	65.0 (32.9)

PET, positron emission tomography; SD, standard deviation; BPAQ, Buss-Perry Aggression Questionnaire.

**Table 2:** Significant correlations between the aggression scores and [^11^C]MDL100907 BP_ND_.

Clinical Variable	MNI Coordinate (x, y, z)	Brain Region	Cluster Size (Voxels)	Peak-Level	
				T Value	*p* Value
Aggression scale total score					
Positive correlation	26, 6, −40	Rt. Temporal pole: MTG	25	5.67	<0.0001
	−32, −10, −32	Lt. FUSI	51	4.89	
	38, −16, −26	Rt. PHG	23	4.45	0.0001
	34, −20, −16	Rt. HIP		4.14	
Physical aggression subscale score					
Positive correlation	−8, 10, −18	Lt. OIF	30	5.25	<0.0001
	−8, 50, −22	Lt. Orbital part of the SFG	26	5.13	
	4, 34, −8	Rt. ACIN	34	4.66	
	−2, 30, −12	Lt. Medial orbital part of the SFG		4.2	0.0001
	−4, 38, −18	Lt. GR		3.88	0.0003
	−44, −56, −10	Lt. ITG	27	4.64	<0.0001
	−48, −48, −6	Lt. MTG		4.63	
	−44, −68, 32	Lt. AG	20	4.54	0.0001
Verbal aggression subscale score					
Positive correlation	8, 60, 10	Rt. Medial part of the SFG	23	6.3	<0.0001
	−32, −12, −32	Lt. FUSI ^†^	51	6.17	
	−22, 62, 6	Lt. SFG	71	5.46	
	28, 8, −40	Rt. Temporal pole: MTG (1)	21	4.92	
	42, 6, −38	Rt. Temporal pole: MTG (2)	23	4.6	
	30, 62, 8	Rt. SFG	30	4.52	0.0001

These results are presented at a peak-level threshold of uncorrected *p* < 0.001 with an extent threshold of 20 voxels. ^†^ Additionally, the verbal aggression subscale score was significantly positively correlated with [^11^C]MDL100907 BP_ND_ in the left FUSI at a peak-level threshold of FDR-corrected *p* < 0.05 with an extent threshold of 10 voxels (FDR-corrected *p* = 0.028, extent threshold k = 12 voxels). BP_ND_, binding potential with respect to non-displaceable compartment; MNI, Montreal Neurological Institute; Rt, right; MTG, middle temporal gyrus; Lt, left; FUSI, fusiform gyrus; PHG, parahippocampal gyrus; HIP, hippocampus; OLF, olfactory cortex; SFG, superior frontal gyrus; ACIN, anterior cingulate and paracingulate gyri; GR, gyrus rectus; ITG, inferior temporal gyrus; AG, angular gyrus; FDR, false discovery rate.

## Data Availability

The data presented in this study are available upon reasonable request from the corresponding authors.

## Supplementary Materials

The following supporting information can be downloaded at: https://www.mdpi.com/article/10.3390/ijms242115697/s1.
