# Supplementary material for: In Vivo Serotonin 5-HT2A Receptor Availability and Its Relationship with Aggression Traits in Healthy Individuals: A Positron Emission Tomography Study with C-11 MDL100907
Source: Int J Mol Sci. 2023 Oct 28;24(21):15697. doi: 10.3390/ijms242115697 (PMC10647245; doi:10.3390/ijms242115697)
Supplement: Supplementary file 1 [file ijms-24-15697-s001.zip › ijms-2685990-supplementary.pdf]

Supplementary Materials

Supplementary Table S1. Sex differences in aggression traits

**Aggression traits**	**Men (n = 10)**	**Women (n = 23)**	***p* value**
Physical aggression	15.2 (2.9)	12.4 (1.9)	0.002*
Verbal aggression	10.5 (4.0)	8.4 (1.9)	0.143
Anger	9.1 (1.4)	9.7 (2.6)	0.468
Hostility	10.8 (2.1)	10.8 (2.3)	0.984
Total	45.6 (6.9)	41.4 (6.1)	0.085

The aggression scores were indicated as mean (standard deviation), and p values were calculated using independent samples t-tests. An asterisk presents statistical significance at $p < 0.05$.

Supplementary Table S2. Results of voxel-based correlation analysis between [^{11}C]MDL100907 BP_{ND} values and age

Correlation	MNI coordinate (x, y, z)	Brain region	Cluster size (voxels)	T value	*p* value
Negative correlation	2, 28, 48	Right superior frontal gyrus (medial part)	1137	5.79	<0.001
	-2, 52, 26	Left superior frontal gyrus (medial part)		5.37	
	6, 22, 56	Right supplementary motor area		4.83	
	-54, -36, 50	Left inferior parietal gyrus	192	5.51	
	-44, -30, 54	Left postcentral gyrus		3.97	
	-42, -42, 58			3.28	0.001
	48, -44, 52	Right inferior parietal gyrus	522	5.37	<0.001
	48, -26, 52	Right postcentral gyrus		5.13	
	56, -18, 16	Right Rolandic operculum		4.33	
	-46, 40, -8	Left inferior frontal gyrus (orbital part)	56	5.23	
	34, 22, -14	Right inferior frontal gyrus (orbital part)	301	5.09	
	48, 18, 2	Right inferior frontal gyrus (triangular part)		4.9	
	40, 12, -14	Right insula		4.62	
	0, -28, 38	Left median cingulate and paracingulate gyri	504	4.92	
	4, -34, 50	Right median cingulate and paracingulate gyri		4.38	
	0, -26, 46	Left median cingulate and paracingulate gyri		4.22	
	-22, 4, -18	Left olfactory cortex	51	4.89	
	-20, -42, 66	Left postcentral gyrus	314	4.82	
	-34, -62, 54	Left superior parietal gyrus		4.56	
	-34, -46, 62			4.29	
	-58, -22, -22	Left inferior temporal gyrus	27	4.78	
	-30, 22, 50	Left middle frontal gyrus	263	4.76	
	-16, 30, 54	Left superior frontal gyrus		4.28	
	-30, 4, 56	Left middle frontal gyrus		4.01	
	-38, 16, -20	Left temporal pole: superior temporal gyrus	118	4.72	
	-38, 20, -12	Left inferior frontal gyrus (orbital part)		3.96	

	-28, 58, 8	Left middle frontal gyrus	121	4.57	
	-32, 58, -2	Left superior frontal gyrus (orbital part)		4.22	
	-30, 54, 18	Left middle frontal gyrus		4.13	
	42, 46, 14	Right middle frontal gyrus	87	4.55	
	40, 44, 24			3.53	0.001
	18, -24, 68	Right precentral gyrus	48	4.53	<0.001
	22, -14, 66	Right superior frontal gyrus		3.32	0.001
	10, -28, 68	Right paracentral lobule		3.22	
	32, -68, 40	Right superior occipital gyrus	57	4.53	<0.001
	38, 52, -4	Right middle frontal gyrus (orbital part)	111	4.52	
	30, 60, -2	Right superior frontal gyrus (orbital part)		4.2	
	-12, 50, 42	Left superior frontal gyrus	33	4.51	
	-18, 46, 38			3.78	
	36, -28, -20	Right fusiform gyrus	33	4.5	
	-38, 22, 42	Left middle frontal gyrus	130	4.45	
	-36, 34, 32			3.77	
	-46, 14, 42			3.55	0.001
	34, 20, 48	Right middle frontal gyrus	60	4.38	<0.001
	36, 14, 42			3.64	
	50, -10, 42	Right precentral gyrus	43	4.36	
	56, 24, 12	Right inferior frontal gyrus (triangular part)	189	4.32	
	44, 26, 26			4.24	
	56, 16, 28	Right inferior frontal gyrus (opercular part)		4.05	
	-28, -26, 70	Left precentral gyrus	161	4.3	
	-22, -24, 62			4.04	
	-16, -32, 74	Left postcentral gyrus		3.73	
	52, 6, 40	Right precentral gyrus	21	4.28	
	36, -28, 66		26	4.21	
	-38, -10, 60	Left precentral gyrus	76	4.21	
	-18, -6, 66	Left supplementary motor area		3.87	
	-32, -10, 66	Left precentral gyrus		3.2	0.002
	54, -60, -14	Right inferior temporal gyrus	73	4.2	<0.001

	60, -50, 0	Right middle temporal gyrus		3.66	
	6, -40, 40	Right Median cingulate and paracingulate gyri	55	4.18	
	-58, 6, 18	Left inferior frontal gyrus (opercular part)	106	4.18	
	-54, 16, 8	Left inferior frontal gyrus (triangular part)		4.16	
	-50, 14, -4	Left temporal pole: superior temporal gyrus		3.77	
	-24, -62, 58	Left superior parietal gyrus	94	4.16	
	-20, -66, 48			3.74	
	-22, -56, 50			3.58	0.001
	-16, 56, 26	Left superior frontal gyrus	20	4.15	<0.001
	40, 36, 36	Right middle frontal gyrus	21	4.11	
	28, -42, -6	Right parahippocampal gyrus	44	4.1	
	24, -50, -6	Right lingual gyrus		3.52	0.001
	2, -56, 40	Right precuneus	56	4.07	<0.001
	0, -64, 46	Left precuneus		3.83	
	22, 48, 36	Right superior frontal gyrus	131	4.06	
	20, 54, 30			3.95	
	12, 48, 38	Right superior frontal gyrus (medial part)		3.53	0.001
	42, 38, -6	Right inferior frontal gyrus (orbital part)	25	4.03	<0.001
	-10, -72, 48	Left precuneus	37	3.98	
	32, -38, 44	Right supramarginal gyrus	103	3.97	
	38, -52, 56	Right superior parietal gyrus		3.59	0.001
	30, -46, 62	Right postcentral gyrus		3.35	
	-44, -2, 2	Left Rolandic operculum	39	3.96	<0.001
	-46, 4, -8	Left superior temporal gyrus		3.8	
	42, -68, -10	Right inferior occipital gyrus	34	3.92	
	22, -34, 66	Right postcentral gyrus	73	3.9	
	24, -26, 68	Right precentral gyrus		3.72	
	-42, -22, 4	Left superior temporal gyrus	36	3.88	
	-42, -14, 10	Left Heschl's gyrus		3.08	0.002
	48, -46, 14	Right superior temporal gyrus	81	3.1	
	-32, 46, 0	Left middle frontal gyrus	34	3.85	<0.001

	-38, 52, -4	Left middle frontal gyrus (orbital part)		3.36	0.001
	-2, -54, 60	Left precuneus	70	3.85	<0.001
	6, -52, 50	Right precuneus		3.38	0.001
	2, -66, 56			3.19	0.002
	-58, -10, 20	Left postcentral gyrus	47	3.8	<0.001
	-62, -16, 30			3.12	0.002
	-56, -38, -16	Left inferior temporal gyrus	43	3.48	0.001
	26, 40, 42	Right middle frontal gyrus	24	3.79	<0.001
	-34, -30, 46	Left postcentral gyrus	27	3.79	
	-58, -30, 44	Left inferior parietal gyrus	45	3.78	
	-58, -32, 36	Left supramarginal gyrus		3.55	0.001
	16, -66, 50	Right superior parietal gyrus	32	3.76	<0.001
	10, -68, 42	Right precuneus		3.23	0.001
	-14, 12, 64	Left supplementary motor area	56	3.66	<0.001
	-20, 8, 56	Left superior frontal gyrus		3.53	0.001
	-46, 28, 26	Left inferior frontal gyrus (triangular part)	21	3.64	<0.001
	64, -20, -18	Right middle temporal gyrus	21	3.63	
	-44, -42, -16	Left inferior temporal gyrus	33	3.57	0.001
	0, 58, -10	Left superior frontal gyrus (medial orbital part)	26	3.49	
	-4, 60, -4	Left superior frontal gyrus (medial orbital part)	27	3.45	
	0, 62, 4	Left superior frontal gyrus (medial part)		3.03	0.002
	-10, 36, 50	Left superior frontal gyrus (medial part)	21	3.31	0.001

These results were obtained at a peak-level threshold of uncorrected $p < 0.005$ with an extent threshold of 20 voxels. BP_{ND}, binding potential with respect to non-displaceable compartment; MNI, Montreal Neurological Institute.

Supplementary Table S3. Sex differences in $[^{11}C]$MDL100907 BP_{ND}

Contrast	MNI coordinate (x, y, z)	Brain region	Cluster size (voxels)	T value	*p* value
Men > Women	-40, -2, 50	Left precentral gyrus	53	5.26	<0.001
	-34, -10, 46			3.22	0.001
	56, -60, 24	Right angular gyrus	40	4.42	<0.001
	52, -60, 32			3.6	0.001
	38, 12, 56	Right middle frontal gyrus	21	4.25	<0.001
	32, 12, -4	Right putamen	21	3.98	
	44, -68, 8	Right middle temporal gyrus	45	3.96	
	-50, 6, 38	Left precentral gyrus	32	3.94	
	-52, 4, 30			3.51	0.001
	-34, -18, -26	Left fusiform gyrus	24	3.6	
Women > Men	4 -94 10	Right calcarine gyrus	45	4.43	<0.001
	4 -90 24	Right cuneus		2.84	0.004

These results were presented at a peak-level threshold of uncorrected $p < 0.005$ with an extent threshold of 20 voxels. BP_{ND}, binding potential with respect to non-displaceable compartment; MNI, Montreal Neurological Institute.

The region of interest (ROI)-based correlation analysis

Based on parameter estimation by kinetic modeling implemented in PMOD software v4.2 (PMOD Technologies Ltd., Zürich, Switzerland), the binding potential with respect to non-displaceable compartment (BP_{ND}) values of [^{11}C]MDL100907 were obtained using a basis function implementation of the simplified reference tissue model [1,2] with cerebellar gray matter as the reference region. In the [^{11}C] MDL100907 BP_{ND} calculation step, regional time-activity curves were extracted from spatially normalized positron emission tomography frames by averaging all voxels within automated anatomical labeling (AAL) [3] atlas-based brain regions containing clusters that showed statistical significance in voxel-based correlation analysis. These AAL atlas-based brain regions included the bilateral superior frontal gyrus (SFG), medial part of right SFG, orbital part of left SFG, medial orbital part of left SFG, left gyrus rectus, left olfactory gyrus, left middle temporal gyrus (MTG), left inferior temporal gyrus, left angular gyrus, left fusiform gyrus, temporal pole of right MTG, right anterior cingulate and paracingulate gyri, right hippocampus, and right parahippocampal gyrus.

To complement the voxel-based results on the relationship between aggression trait and [^{11}C] MDL100907 BP_{ND} in specific brain regions, we also performed an ROI-based correlation analysis with age and sex as covariates with the same data using the Statistical Package for the Social Sciences v28.0 (IBM Corp., Armonk, NY, USA). Significant results were identified at a threshold of two-tailed $p < 0.05$.

References

1. Lammertsma, A.A.; Hume, S.P. Simplified Reference Tissue Model for PET Receptor Studies. *NeuroImage* **1996**, *4*, 153–158, doi:10.1006/nimg.1996.0066.
2. Gunn, R.N.; Lammertsma, A.A.; Hume, S.P.; Cunningham, V.J. Parametric Imaging of Ligand-Receptor Binding in PET Using a Simplified Reference Region Model. *NeuroImage* **1997**, *6*, 279–287, doi:10.1006/nimg.1997.0303.
3. Tzourio-Mazoyer, N.; Landeau, B.; Papathanassiou, D.; Crivello, F.; Etard, O.; Delcroix, N.; Mazoyer, B.; Joliot, M. Automated Anatomical Labeling of Activations in SPM Using a Macroscopic Anatomical Parcellation of the MNI MRI Single-Subject Brain. *NeuroImage* **2002**, *15*, 273–289, doi:10.1006/nimg.2001.0978.

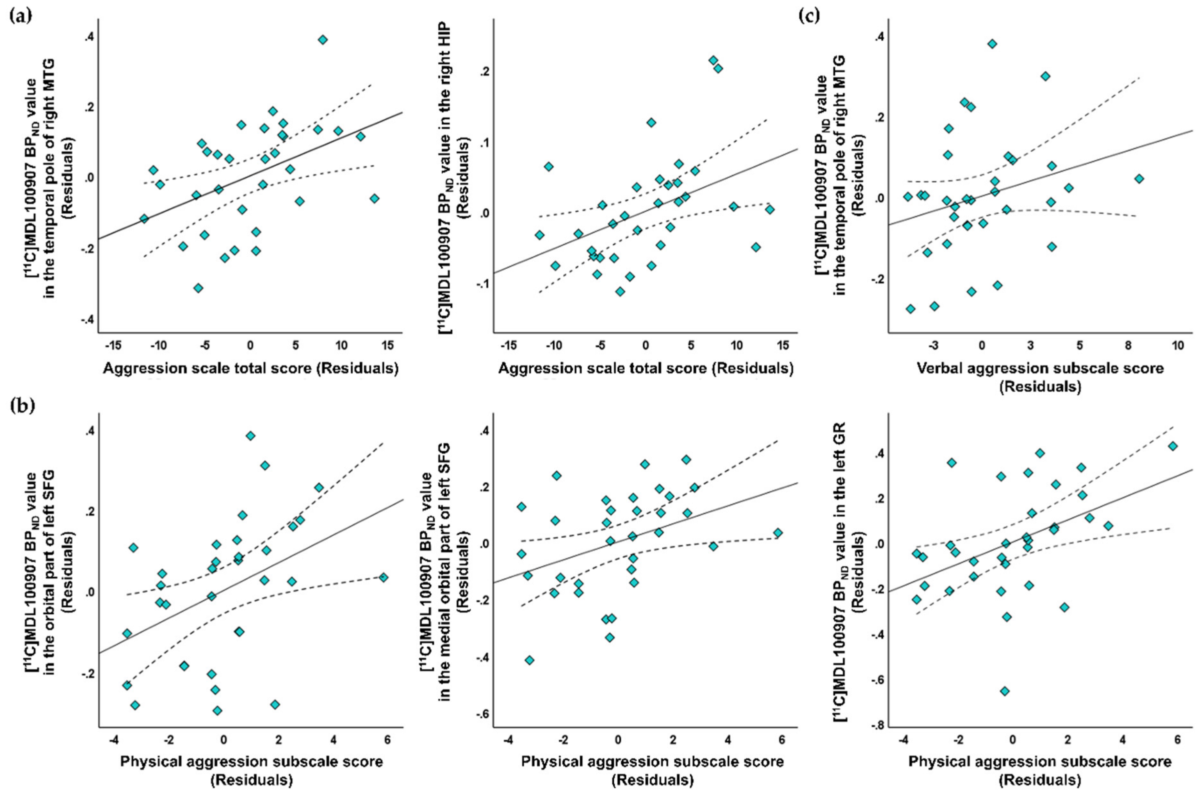

Supplementary Figure S1. Scatter plots showing the ROI-based partial correlations between the aggression scores and [^{11}C]MDL100907 BP_{ND} in AAL atlas-based brain regions. (a) The aggression scale total score had significant positive correlations with [^{11}C]MDL100907 BP_{ND} in the temporal pole of right MTG (r = 0.447, p = 0.012) and right HIP (r = 0.429, p = 0.016). (b) The physical aggression subscale score had significant positive correlations with [^{11}C]MDL100907 BP_{ND} in the orbital part of left SFG (r = 0.425, p = 0.017), medial orbital part of left SFG (r = 0.387, p = 0.032), and left GR (r = 0.444, p = 0.012). (c) The verbal aggression subscale score had a significant positive correlation with [^{11}C]MDL100907 BP_{ND} in the temporal pole of right MTG (r = 0.382, p = 0.034). The mint rhombi represent ordered pairs of the unstandardized residuals calculated from two separate linear regressions of the aggression scores and [^{11}C]MDL100907 BP_{ND} in specific brain regions in regard to age and sex. The solid and dotted lines indicate the regression lines and 95% confidence intervals, respectively. ROI, region of interest; BP_{ND}, binding potential with respect to non-displaceable compartment; AAL, automated anatomical labeling; MTG, middle temporal gyrus; HIP, hippocampus; SFG, superior frontal gyrus; GR, gyrus rectus.
